# Increased sucrose consumption in mice gene-targeted for *Vmat2* selectively in NeuroD6-positive neurons of the ventral tegmental area

**DOI:** 10.3389/fnmol.2023.1069834

**Published:** 2023-02-07

**Authors:** Zisis Bimpisidis, Gian Pietro Serra, Niclas König, Åsa Wallén-Mackenzie

**Affiliations:** Unit of Comparative Physiology, Department of Organismal Biology, Uppsala University, Uppsala, Sweden

**Keywords:** dopamine, NeuroD6, VTA, sucrose, VMAT2, motivation, self-administration

## Abstract

Ventral tegmental area (VTA) dopamine (DA) neurons are implicated in reward processing, motivation, reward prediction error, and in substance use disorder. Recent studies have identified distinct neuronal subpopulations within the VTA that can be clustered based on their molecular identity, neurotransmitter profile, physiology, projections and behavioral role. One such subpopulation is characterized by expression of the *NeuroD6* gene, and projects primarily to the nucleus accumbens medial shell. We recently showed that optogenetic stimulation of these neurons induces real-time place preference while their targeted deletion of the *Vmat2* gene caused altered response to rewarding substances, including ethanol and psychostimulants. Based on these recent findings, we wanted to further investigate the involvement of the NeuroD6-positive VTA subpopulation in reward processing. Using the same *NeuroD6^Cre+/wt^*;*Vmat2^flox/flox^* mice as in our prior study, we now addressed the ability of the mice to process sucrose reward. In order to assess appetitive behavior and motivation to obtain sucrose reward, we tested conditional knockout (cKO) and control littermate mice in an operant sucrose self-administration paradigm. We observed that cKO mice demonstrate higher response rates to the operant task and consume more sucrose rewards than control mice. However, their motivation to obtain sucrose is identical to that of control mice. Our results highlight previous observations that appetitive behavior and motivation to obtain rewards can be served by distinct neuronal circuits, and demonstrate that the NeuroD6 VTA subpopulation is involved in mediating the former, but not the latter. Together with previous studies on the NeuroD6 subpopulation, our findings pinpoint the importance of unraveling the molecular and functional role of VTA subpopulations in order to better understand normal behavior and psychiatric disease.

## Introduction

The midbrain dopamine (DA) system has been implicated in reinforcement learning, reward prediction error (Schultz et al., 1997), motivation (Berke, 2018), incentive salience (Berridge and Robinson, 1998) and in diseases where these functions are compromised, such as substance use disorder (Lüscher, 2016). Expanding on the classical classification of DA circuits into nigrostriatal and mesolimbic systems (Björklund and Dunnett, 2007), recent studies have focused on the heterogeneity of DA neurons within the midbrain, in terms of gene expression, projection patterns and behavioral role (Chung et al., 2005; Greene et al., 2005; Lammel et al., 2008, 2011, 2012; Poulin et al., 2014, 2018; Viereckel et al., 2016; Bimpisidis et al., 2019; Heymann et al., 2020; König et al., 2020; Serra et al., 2021). Characterizing differences between previously considered homogeneous DA populations will lead in deciphering the functional role of distinct subpopulations, make it possible to selectively target them for treatment of disease and eventually avoiding unwanted side effects that might occur by targeting the DA system as a whole.

It is increasingly understood that DAergic systems can mediate distinct aspects of behavior depending on the target projection area. Substantia nigra pars compacta (SNc) and ventral tegmental area (VTA) DA neurons have different topographical projections that in turn mediate diverse aspects of reward-related behavior. For example, incentive value attribution is mediated by nucleus accumbens (NAc) core projecting DA neurons and not by those projecting to the NAc shell (NAcSh; Saunders et al., 2018). Within the SNc, separate DA neuron groups project to the dorsolateral (DLS) and to the dorsomedial striatum (DMS), have distinct electrophysiological properties, and respond differently to reward delivery and aversive stimuli. Those neurons projecting to DMS reduce, while those projecting in DLS increase their activity in response to foot shock (Lerner et al., 2015).

Regarding the behavioral role of the VTA, recent fiber photometry studies have demonstrated that the activity of DA neurons in the medial and lateral parts is correlated with reward prediction error and salience, respectively, and the overall aftermath is dependent on the temporal scale of activation of these subareas (Cai et al., 2020). Sophisticated analysis of complex behavioral data and Ca^2+^ transients of VTA DA neurons showed that these neurons form clusters that respond more strongly to specific aspects of a reward task (sensory, motor, cognitive), a function related to anatomical location of the cluster. However, neurons in each cluster do not seem to be “specialized” in only one behavioral variable but to respond to more than one (Engelhard et al., 2019).

The specific input–output circuitry characteristics of subgroups of VTA DA neurons defines their profile in terms of behavioral role (Lammel et al., 2011, 2012; de Jong et al., 2019). Separate subpopulations within the VTA receive different inputs and mediate opposite types of behavior, rewarding or aversive. A rewarding stimulus such as cocaine, affects mostly medial VTA DA neurons projecting to the medial NAcSh (mNAcSh) while aversive stimuli like foot shocks are processed by those DA neurons projecting to the medial prefrontal cortex (mPFC). VTA DA neurons connected to the NAc lateral shell respond to both types of stimuli in a similar way (Lammel et al., 2011).

Exploiting the unique gene expression patterns of different neuronal subtypes has been recently employed to unravel the projection patterns and role in behavior of distinct neuronal subpopulations within the VTA (Bimpisidis et al., 2019; Heymann et al., 2020; König et al., 2020; Kramer et al., 2021; Serra et al., 2021). For example, Heymann and colleagues (Heymann et al., 2020) used genetic and viral approaches to target VTA neurons characterized by specific expression of peptides. They showed that VTA neurons expressing *Crhr1* project selectively to the NAc core and those expressing *Cck* to the mNAcSh. Behaviorally, activation of VTA-NAc core neurons is sufficient to promote acquisition of an instrumental behavior while VTA-NAc shell activation is responsible for maintaining an instrumental response. However, the two different subpopulations act in synergy to optimize behavior (Heymann et al., 2020). More recently, Serra and colleagues, using conditioned knock-out (cKO) approaches, showed that the medially located VTA DA population characterized by the expression of *TrpV1* is involved in modulating amphetamine-induced locomotion (Serra et al., 2021).

A recently described VTA subpopulation is characterized by the expression of the *NeuroD6* gene (also known as *NEX1M*). The gene is selectively expressed within subsets of VTA DA neurons but not in those of the neighboring substantia nigra compacta (SNc; Viereckel et al., 2016; Khan et al., 2017; Kramer et al., 2018). We recently showed that NeuroD6- (or NEX-Cre)-expressing neurons constitute 12% of all VTA tyrosine hydroxylase (TH) positive neurons, are mostly located in the medial nuclei of the VTA and project preferentially to the medial part of the nucleus accumbens shell (mNAcSh). A small percentage of them (12%) co-releases glutamate and optogenetic stimulation of these neurons induces real-time place preference. To address the role of DA released by this VTA subpopulation, we generated a conditional knock-out (cKO) mouse line, created by crossing *NEX^Cre+/wt^* mice and mice having the gene coding for the vesicular monoamine transporter – 2 (*Vmat2*) flanked by LoxP sites (*Vmat2^flox/flox^*; Narboux-Nême et al., 2011). We observed that disruption of NEX-Cre neurons’ ability to release DA renders mice hypersensitive to the locomotor effects of repeated injections of amphetamine and results in altered responses toward ethanol consumption (Bimpisidis et al., 2019).

The mesolimbic DAergic system is involved in processing both drug and natural rewards (Di Chiara and Bassareo, 2007; Ikemoto, 2007). It remains to be answered whether the NeuroD6 DA subpopulation is involved in reinforcement learning and motivation for food reward. To answer this question, we used the cKO mouse line generated and characterized previously (Bimpisidis et al., 2019) to ablate DA release selectively from NeuroD6-expressing neurons. We tested cKO and littermate control mice in an operant sucrose self-administration task consisting of different phases modeling separate behavioral aspects of appetitive behavior. We assessed the consumption of sucrose rewards under fixed ratio schedules of reinforcement, and the motivation to obtain reward using the well-established progressive ratio schedule. We observed that cKO mice nose-poke for, receive and consume/ingest more sucrose rewards than control littermates in fixed ratio schedules. Interestingly, when tested in the progressive ratio schedule, their motivation or will to work for sucrose remained unaltered. Finally, during a cue-induced reinstatement phase of the protocol, cKO mice had higher number of magazine entries, suggesting that the ability of that mice to process cues paired to reward was impaired. Our results add up to the increasing knowledge on the involvement of distinct DA subpopulations in behavior and suggest that separate subcircuits within the VTA might serve the appetitive and motivational aspects of reward-related behaviors. This is of interest in better understanding DA-related diseases such as drug or food addiction and relevant in developing novel therapeutic approaches aiming to target well-defined neuronal subpopulations.

## Materials and methods

### Animals

**Figure 1:**
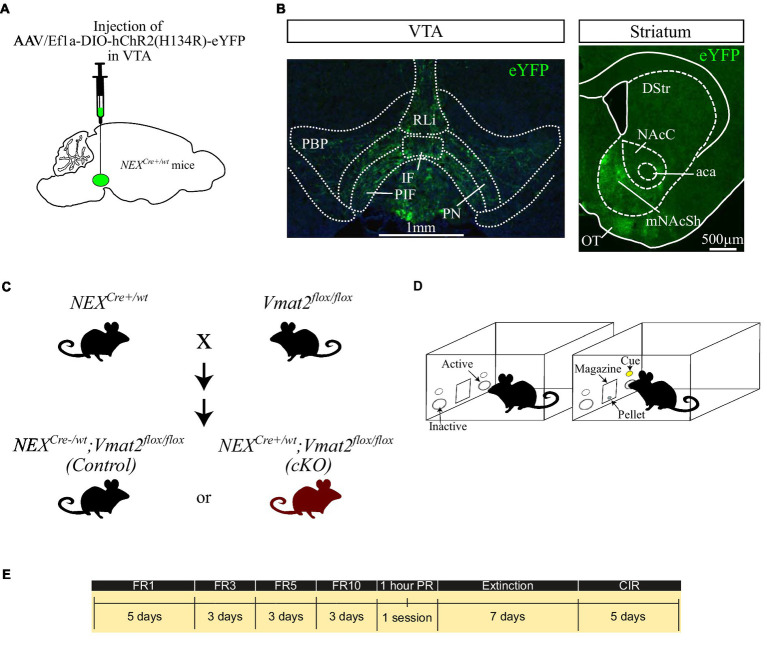
Schematic representation of the virus injection in *NEX^Cre+/wt^* mice **(A)**. Fluorescent images showing the distribution of NEX-Cre neurons in the VTA (**B**, left panel) and their main projections to the mNAcSh (**B**, right). Breeding strategy to obtain *NEX^Cre−/wt^*;Vmat2*^flox/flox^* (Control) and *NEX^Cre+/wt^*;*Vmat2^floxf/lox^* (cKO) mice **(C)**. Schematic of the apparatus **(D)** and the schedule of the behavioral experiments **(E)**. aca: anterior commissure, anterior part, DStr: dorsal striatum, NAcC: nucleus accumbens core, mNAcSh: nucleus accumbens medial shell, IF: interfascicular nucleus, OT: olfactory tubercule, PBP: parabranchial pigmented nucleus, PIR: parainterfascicular nucleus, PN: paranigral nucleus, RLi: rostral linear nucleus. CIR: Cue-induced reinstatement.

*NEX^Cre−/wt^*;*Vmat2^flox/flox^* (Control) and *NEX^Cre+/wt^*;*Vmat2^flox/flox^* (cKO) were generated as described previously (Bimpisidis et al., 2019) and as depicted in Figure 1C. Briefly, *NeuroD6/NEX^Cre+/wt^* transgenic mice (Goebbels et al., 2006) were bred with *Vmat2^flox/flox^* mice, in which exon 2 of the Vmat2 gene is flanked by LoxP sites (Narboux-Nême et al., 2011) to generate cKO mice in which Vmat2 exon 2 is ablated on NEX-Cre-mediated recombination of LoxP sites. VMAT2 is responsible for concentrating monoamines in synaptic vesicles and thus for their release in the synaptic cleft; abnormal expression of the transporter leads to impaired neurotransmission (Narboux-Nême et al., 2011). Littermates negative for NEX-Cre served as controls (Control). Mice were genotyped by PCR as described previously (Bimpisidis et al., 2019) with the following primers: Cre: 5’-ACG AGT GAT GAG GTT CGC AAG A-3′; 5’-ACC GAC GAT GAA GCA TGT TTA G-3′; Vmat2Lox: 5’-GAC TCA GGG CAG CAC AAA TCT CC-3′; 5′-GAA ACA TGA AGG ACA ACT GGG ACC C-3′. All animals were housed on a standard 12 h sleep/wake cycle (7:00 A.M. lights on, 7:00 P.M. lights off) and housed according to Swedish (Animal Welfare Act SFS 1998:56) and European Union legislation (Convention ETS 123 and Directive 2010/63/EU). Mice were food restricted (85% of initial body weight) throughout the experiments. All experiments were conducted with permission from Uppsala University Ethical Committee for Use of Animals.

### Stereotaxic injections

AAV5-EF1a-DIO-ChR2 (H134)-eYFP virus was purchased from University of North Carolina, Vector Core Facilities, and delivered to the VTA by stereotaxic surgery as previously described (Bimpisidis et al., 2019, 2020; Figure 1A) in order to visualize VTA NEX-Cre positive neurons and their projections. Briefly, *NEX^Cre+/wt^* mice (>8 weeks old; >20 g) were deeply anesthetized with isoflurane and received 300 nl of virus in the right VTA (AP: −3.45 mm, L: −0.2 mm, V:-4.4 mm according to Franklin and Paxinos, 2008) at 100 nl min^−1^ flow rate. Four weeks after injection the mice were transcardially perfused, their brains were collected and cut in a vibratome at 30um-thick sections. The sections were mounted, coverslipped and imaged using a Leica epifluorescent microscope.

### Operant apparatus

Instrumental testing was performed in operant chambers (Med Associates Inc., Fairfax, United States) equipped with nosepoke devices on each side of a food magazine. Nose-poking to the active nosepoke (right) activated a cue light above the nose poke and a pellet dispenser which delivered a 20 mg sucrose pellet (5TUT, TestDiet, St. Louis, United States; Figure 1D) according to the different phases of the task.

### Sucrose self-administration paradigm

Operant sucrose Self-Administration (SA) was performed as described previously (Alsiö et al., 2011). We used operant conditioning to assess incentive-guided behaviors similarly to what has been used to study addiction-related behaviors. Thus, we included Fixed Ratio (FR) schedules of reinforcement to measure sucrose “taking” or consumption, a Progressive Ratio (PR) schedule of reinforcement to express quantitively the motivation to obtain sucrose, an extinction phase and a final cue-induced reinstatement (CIR) phase to model sucrose seeking and/or the efficacy of sucrose-related cues to elicit instrumental responses (Roberts et al., 1989; Epstein et al., 2006; Grimm and Sauter, 2020; Tsibulsky and Norman, 2021).

The timeline of the experiments is depicted in Figure 1E. At the first phase of the task, food restricted mice were placed in the chambers under a FR1 schedule in which each active nose poke resulted in the delivery of 1 sucrose pellet. The learning criterion was met if the mouse obtained ≥10 rewards and then having stable responses in terms of active nose pokes (<15% difference between sessions) for 3 consecutive days. For FR1, 2 days before the aforementioned criterion was met were included in the graph and analysis. The mice were then moved to FR3, where 3 active nose pokes resulted the delivery of 1 reward. When mice demonstrated stable responses in the active nose poke (<15% difference between sessions) for 3 days were moved to increased ratios, firstly to FR5 and finally to FR10. The FR sessions were followed by a single, one-hour PR session where the increase in number of responses required to obtain each pellet during the sessions was increased and calculated according to the formula 5e^(reinforcer number x 0.2)^-5, rounded to the nearest integer (Richardson and Roberts, 1996). As breaking point was considered the last number of poke requirement before the session end. After the PR session the mice underwent 7 days of extinction where nose-poking did not result in any sucrose delivery or cue-presentation. The experiment was finalized with 5-days of CIR sessions. During this phase each nose poke resulted in cue-presentation but not sucrose pellet delivery. All sessions except PR lasted for 30 min.

### Statistical analysis

All data are expressed as mean ± SEM and were analyzed with GraphPad 8.0 (RRID:SCR_002798). Statistical significance was set at *p* < 0.05 and details on tests can be found in the “Results” sections and/or in figure legends.

## Results

### NEX-Cre neurons are confined within the VTA and project mainly to the nucleus accumbens medial shell

Brain tissue analysis of *NEX^Cre+/wt^* mice injected with optogenetic viruses confirmed that NEX-Cre neurons are located within the medial aspects of the VTA. The majority were located in the paranigral, parainterfascicular and parabranchial pigmented nuclei, while smaller numbers were scattered in the interfascicular and rostral linear nuclei (Figure 1B, left panel). The strongest projections of eYFP labeled neurons was observed in the mNAcSh (Figure 1B, right panel), in accordance with previous studies (Bimpisidis et al., 2019; Kramer et al., 2021).

### *NEX^Cre+/wt^*;*Vmat2^flox/flox^* mice demonstrate more consummatory behavior than their wild-type littermates

**Figure 2:**
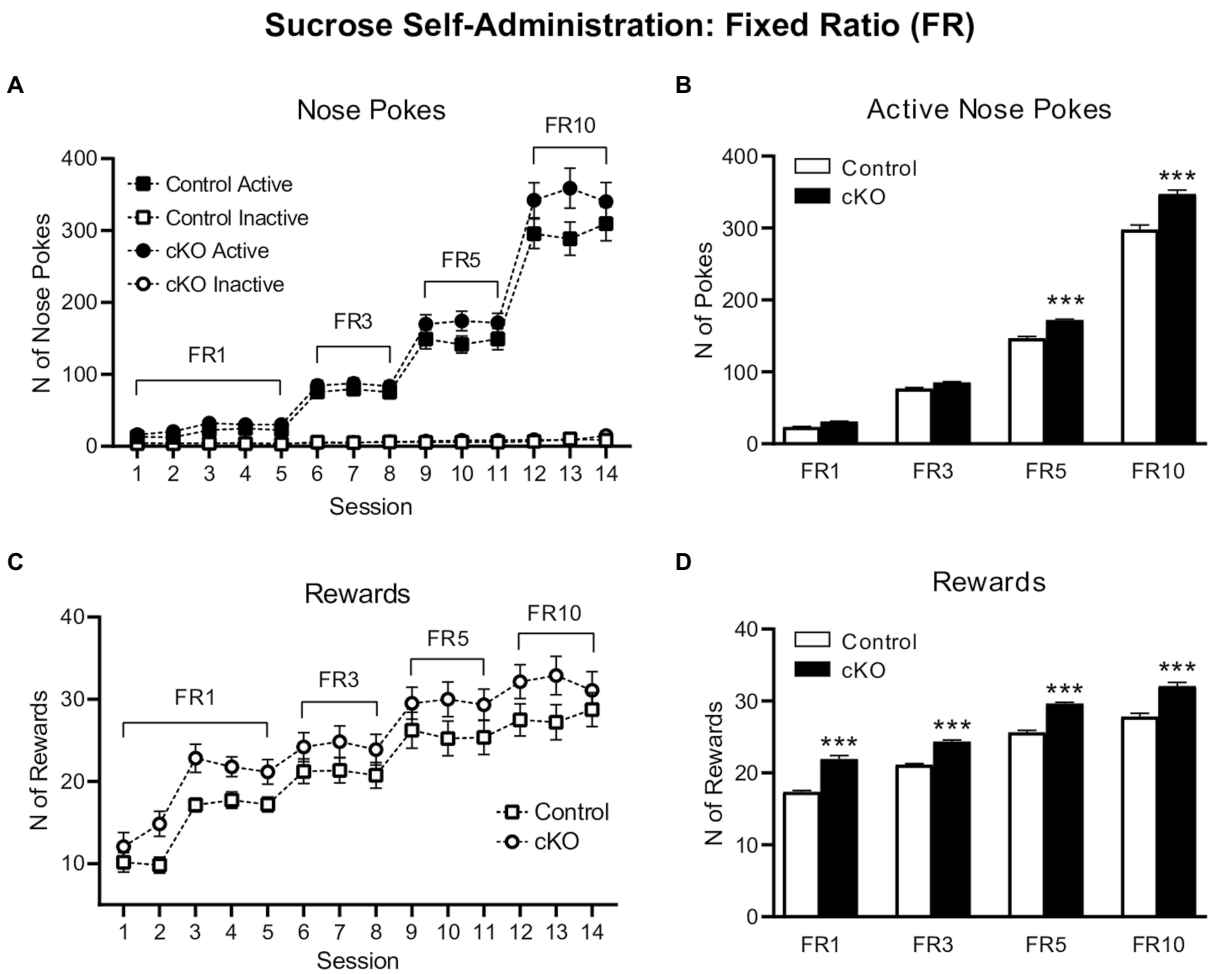
Active (filled symbols) and inactive (clear symbols) nose pokes of control (squares) and cKO (circles) mice throughout the Fixed Ratio (FR) sessions **(A)**. Average of active nose pokes of control (white bars) and cKO (black bars) mice in FR1, FR3, FR5 and FR10 sessions **(B)**. Rewards obtained by control (squares) and cKO (circles) throughout the Fixed Ratio (FR) sessions **(C)**. Average of rewards gained by control (white bars) and cKO (black bars) mice in FR1, FR3, FR5 and FR10 sessions **(D)**. Data are expressed as mean ± SEM. ***p < 0.001 Sidak’s multiple comparisons test vs. control mice.

The first phase of the operant sucrose SA experiment consisted of increasing FR schedules of reinforcement and when met, a single reward was delivered in the food magazine. cKO mice poked the active nose-poke more than controls throughout this phase of the task, with greater differences revealed with increasing ratio demands [session x genotype x nose poke effect, *F*_(13,1,338)_ = 2.195, *p* = 0.0081, mixed effects model; Figure 2A]. Two-way ANOVA analysis on the average active nose pokes for each schedule of reinforcement (Figure 2B) revealed significant effects of schedule [*F*_(3,204)_ = 4,450, *p* < 0.0001], genotype [*F*_(1,204)_ = 146.3, *p* < 0.0001] and schedule x genotype interaction [F_(3,204)_ = 24.99, *p* < 0.0001]. Sidak’s multiple comparisons test showed that cKO mice displayed a trend toward increased average numbers of active nose pokes for FR1 and FR3 schedules compared to controls (*p* = 0.0751 and *p* = 0.0669, respectively), that reached statistical significance during FR5 (*p* < 0.0001) and FR10 (*p* < 0.0001; Average active nose pokes for each schedule of reinforcement: cKO FR1: 30.92 ± 0.67, N = 34; Control FR1: 23.29 ± 0.77, N = 34; cKO FR3: 85.18 ± 1.18, N = 26; Control FR3: 76.76 ± 1.45, N = 29; cKO FR5: 172 ± 1.295, N = 21; Control FR5: 146.5 ± 2.54, N = 25; cKO FR10: 347 ± 5.93, N = 20; Control FR10: 297.9 ± 6.15, N = 20; Figure 2B).

The increased number of responses on the active nose pokes by the cKO mice was accompanied by a larger number of consumed rewards throughout the 14 sessions of the experiment [effect of session *F*_(13,669)_ = 41.4, *p* < 0.001; effect of genotype *F*_(1,66)_ = 9.56, *p* = 0.003 but not of session x genotype F_(13,669)_ = 0.478, *p* = 0.937, mixed effects model; Figure 2C]. Thus, the average number of rewards obtained by cKO mice was consistently higher than control mice for every FR schedule of reinforcement [Two-way ANOVA, effect of schedule: F_(3,204)_ = 351.3, *p* < 0.0001; genotype F_(1,204)_ = 252.7, *p* < 0.0001; schedule x genotype F_(3,204)_ = 1.607, *p* = 01888. Sidak’s multiple comparisons test on averages: FR1: cKO 21.93 ± 0.48, Control 17.36 ± 0.19, *p* < 0.0001; FR3: cKO 24.31 ± 0.28, Control 21.11 ± 0.18, *p* < 0.0001; FR5: cKO 29.62 ± 0.2, Control 25.61 ± 0.32, *p* < 0.0001; FR10: cKO 32.05 ± 0.52, Control 27.82 ± 0.47, *p* = 0.0001; Figure 2D].

These results indicate that cKO mice, that lack the capacity to release DA from the NEX-Cre positive VTA subpopulation, work more to obtain sucrose and thus demonstrate increased consummatory behavior compared to their wild-type littermates.

### Motivation to obtain sucrose is not altered in *NEX^Cre+/wt^*;*Vmat2^flox/flox^* mice

**Figure 3:**
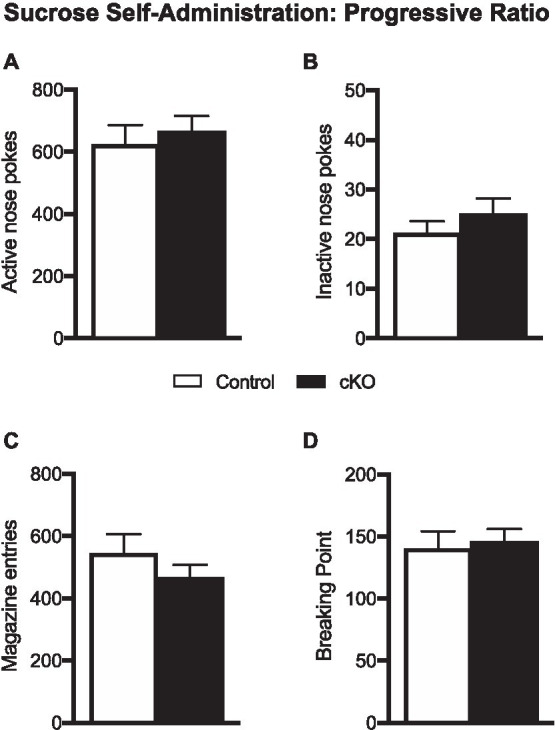
Active **(A)** and inactive pokes **(B)**, magazine entries **(C)** and breaking point **(D)** of control (white bars) and cKO (black bars) mice during the one-hour progressive ratio session. Data are expressed as mean ± SEM.

When tested in a progressive ratio schedule of reinforcement, where the subsequent reward delivery demanded higher-effort poking behavior, cKO mice did not differ on their level of motivation to obtain sucrose from control mice. Thus, cKO and their wild-type littermates had similar number of active nose pokes (cKO: 664.2 ± 51.73, N = 20; Control 622 ± 65.32, N = 20; *p* = 0.615; Figure 3A), inactive nose pokes (cKO: 24.95 ± 3.22; Control 21.1 ± 2.51; *p* = 0.352; Figure 3B), magazine entries (cKO: 464.4 ± 43.29; Control: 543.4 ± 62.95; *p* = 0.308; Figure 3C) and breaking point (cKO 145.6 ± 10.44, Control 140.2 ± 14.17, *p* = 0.758; Figure 3D), a quantitative measurement of motivation to obtain a reward. Unlike consummatory behavior, the motivational processes to obtain a highly salient food reward do not seem to depend on DA released by the NEX-Cre positive VTA subpopulation.

### *NEX^Cre+/wt^*;*Vmat2^flox/flox^* and control mice do not differ in extinction and cue-induced reinstatement of operant behavior in the sucrose SA paradigm

**Figure 4:**
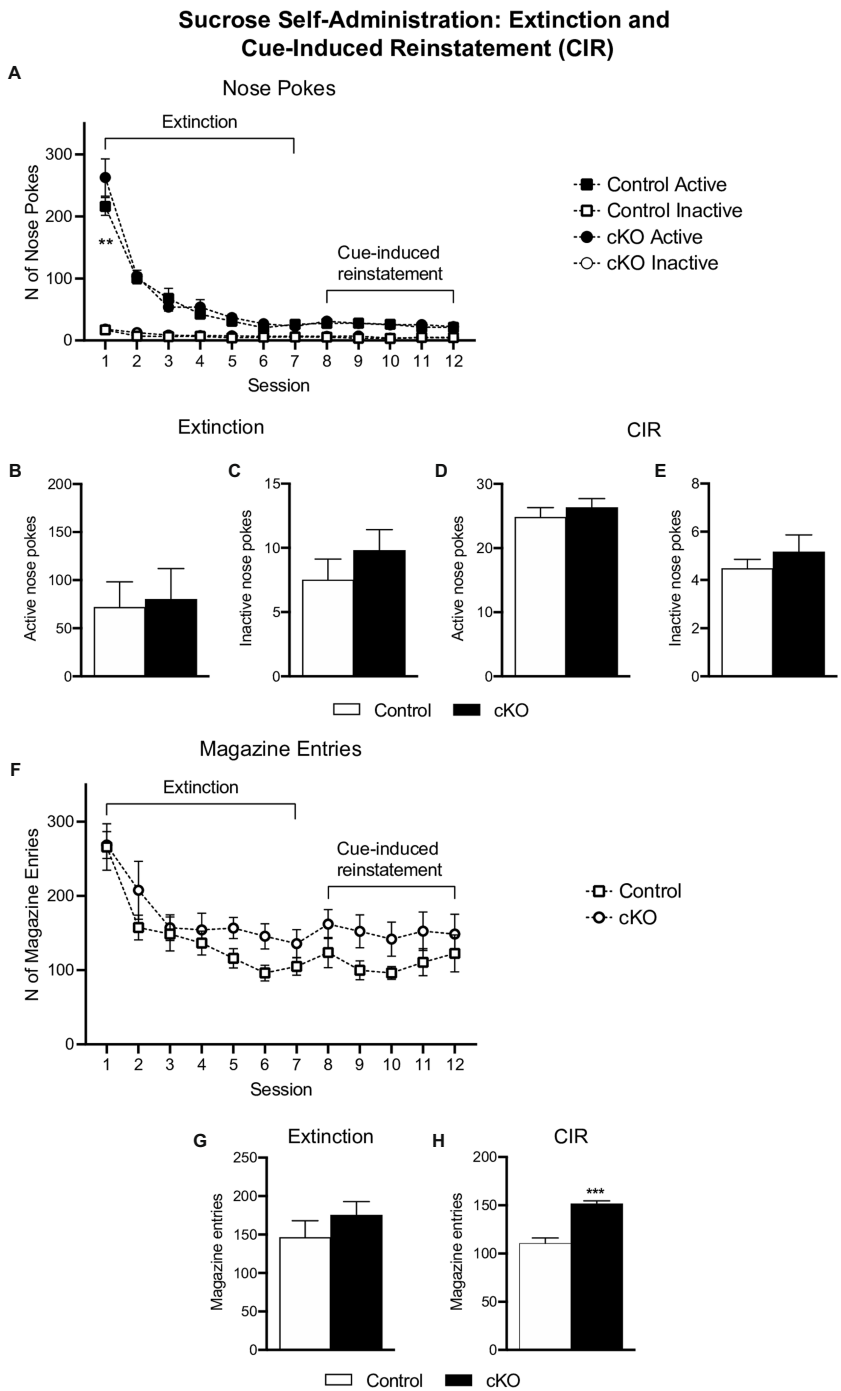
Active (filled symbols) and inactive (clear symbols) nose pokes of control (squares) and cKO (circles) mice throughout the extinction and cue-induced reinstatement (CIR) phases **(A)**. Average of active **(B,D)** and inactive **(C,E)** nose pokes of control (white bars) and cKO (black bars) mice in extinction and CIR phases. Magazine entries throughout the extinction and CIR phases **(F)** and average of responses separate for each phase **(G,H)**. Data are expressed as mean ± SEM. **p = 0.0011, Tukey’s *post-hoc* comparison test vs. control mice; ***p < 0.001 vs. control mice unpaired *t*-test.

After the progressive ratio session, cKO and control mice underwent a seven-day extinction phase, followed by 5 days of CIR. Responding in nose pokes did not differ between genotypes throughout the testing period [effect of session x genotype x nose poke, *F*_(11,432)_ = 1.299, *p* = 0.3192, 3-way ANOVA; Figure 4A]. Similarly, the average of pokes during extinction [active cKO: 80.05 ± 32.06 N = 19, Control:71.99 ± 26.23 N = 19, *p* = 0.849; inactive cKO: 9.79 ± 1.630, Control 7.5 ± 1.612, *p* = 0.339, unpaired *t*-test; Figures 4B,C] and CIR [active cKO: 26.28 ± 1.42, Control:24.83 ± 1.47, *p* = 0.498; inactive cKO: 5.16 ± 0.71, Control 4.47 ± 0.38, *p* = 0.421, unpaired *t*-test; Figures 4D,E] did not differ between genotypes. Furthermore, magazine entries did not differ when analyzed for each session [effect of session x genotype *F*_(11,396)_ = 0.656, *p* = 0.780, 2-way ANOVA; Figure 4F]. The average of magazine entries during extinction was not different between genotypes (cKO: 175 ± 17.78; Control:146.4 ± 21.63, *p* = 0.328, unpaired *t*-test) but was higher in cKO compared to control mice in CIR sessions (cKO: 151.3 ± 3.27; Control:110.5 ± 5.63, *p* < 0.001, unpaired *t*-test; Figures 4G,H). While both genotypes extinguish their operant responses in a similar manner, cKO mice visit the magazine more frequently during CIR, a behavior possibly reflecting abnormal sensitivity to cues.

## Discussion

The gene encoding the transcription factor NeuroD6 is expressed in a relatively small number of DA neurons of the VTA primarily located in paranigral, parainterfascicular and parabranchial pigmented subnuclei of the VTA (Viereckel et al., 2016; Khan et al., 2017; Kramer et al., 2018, 2021; Bimpisidis et al., 2019). Using our previously published cKO approach to target the NeuroD6 DA subpopulation, we here tested cKO mice in an operant sucrose SA task, and compared their performance with that of age-and sex-matched control mice. The operant SA task differs from the sucrose preference test we used before (Bimpisidis et al., 2019) and can reveal different behaviors affected by the given genetic manipulation. Thus, while the later can give information for anhedonia-like symptoms (Liu et al., 2018), the complexity of the former can answer questions on whether consumption, motivation or cue-induced behavior is altered in cKO mice with respect to wt littermate controls. It is possible that knocking-out genes within the DA system can induce alterations in homeostatic feeding behavior. Nevertheless, the fact that cKO mice and littermate controls show no differences in weight (Bimpisidis et al., 2019; current study, data not shown) led us to exclude this possibility. The operant SA task made evident that cKO mice demonstrated more responses and obtained more rewards under several FR schedules compared to controls. This shows that NeuroD6 neurons are involved in regulating consummatory behavior through DA release. On the other hand, motivation to receive sucrose rewards did not differ between cKO and control mice, as measured by a PR schedule, suggesting that the NeuroD6 VTA subpopulation is not involved in motivational aspects related to food reward. Finally, no differences in active and inactive nose pokes were observed during extinction and CIR schedules but cKO mice visited the food magazine more times than their control littermates during this latter phase of the experiment, possibly indicating that cKO mice show abnormal sensitivity to reward-related cues.

In a previous study (Bimpisidis et al., 2019), we described that NEX-Cre neurons project mainly to the mNAcSh and that ablation of Vmat2 from these neurons results in elevated locomotor responses to amphetamine. In the current study we demonstrate that these same cKO mice make more operant responses to obtain sucrose rewards. These results might seem counterintuitive, given the well characterized role of DA in both motor and consummatory behaviors provided by studies targeting the DAergic systems unselectively. A possible explanation of our results could be given by the fact that only a small percentage of VTA DA neurons express *NeuroD6* (about 12%; Bimpisidis et al., 2019) and their inability to release DA might lead to different behavioral outcomes from those expected when larger ablations or disturbances of the DA system take place.

*NeuroD6* is already expressed at E14.5 in cells positive for other dopaminergic markers, indicating that the processes to form a unique DA subpopulation within the VTA begin early in development (Dumas and Wallén-Mackenzie, 2019). Studies on NeuroD6 knock-out mice, have highlighted the importance of the gene in the normal development of the DA system; KO mice show reduced number of midbrain DA neurons (Khan et al., 2017). It is possible that the cKO approach we followed in the current study also affected the maturation of the developing DA system by disrupting DA transmission from NeuroD6+ cells. Furthermore, reduced DA tone from this subpopulation might have induced post-synaptic adaptive changes in other systems, that could explain our findings. For instance, the higher number of operant responding during fixed ratios of reinforcement might reflect increased positive experiences that are mainly non-DA related; indeed, they seem to be mediated by GABAergic and opioidergic systems (Berridge and Robinson, 1998). Possibly, cKO mice work for and consume more sucrose because they experience greater pleasurable effects due to occurrence of developmental changes following the absence of DA release from NeuroD6+ neurons throughout the lifespan, but this is a hypothesis that has to be tested experimentally. A way to verify this hypothesis would be to use inducible Cre lines or viral strategies to target this specific neuronal subpopulation in combination with behavioral tests that assess hedonic reactions (Berridge and Robinson, 1998).

Additional plastic changes, such as the overactivity of the rest, non NeuroD6+, neurons of the DA system cannot be excluded. Different DA subcircuits work in synergy to mediate behavior and transform incentive to actions. In this direction, disruption of DA release toward mNAcShell (and as mentioned throughout development) might render DA target areas more sensitive to DA deriving from intact DA populations and in consequence to enhance response-outcome associations relevant to food reward delivery, or more sensitive to cues related to it. The increases in magazine entries during the CIR phase of the experiment may support this notion, but it requires further investigation.

Manipulations of the DA system affect higher effort schedules of reinforcement rather than actions of lower cost (Salamone et al., 1991; Cheeta et al., 1995; Ikemoto and Panksepp, 1996; Aberman and Salamone, 1999; Reilly, 1999; Barbano and Cador, 2006; Veeneman et al., 2012). In the current study, no changes in performance during higher effort/increasing demand-related schedules of reinforcement were observed when cKO and control mice were tested in the progressive ratio test. Our results suggest that consummatory and motivational aspects of reward-related behavior are served by distinct DA circuits and that the NeuroD6-positive DA neurons are involved in the former and not the latter. A possible limitation of our study design is the relatively short period of the PR sessions. It remains unknown if testing for longer time periods would be sufficient to reveal differences between cKO and control mice. However, given the fact that we already observed significant differences between genotypes during the 30-minute-long FR sessions, it is likely that one-hour sessions of PR testing were appropriate for the scope of our study, and in accordance to the literature.

Newer molecular methods permit the separation and characterization of DA neurons within the midbrain based on their molecular profile, both between SNc and VTA, but also within each region (Poulin et al., 2014, 2018; La Manno et al., 2016; Viereckel et al., 2016; Nagaeva et al., 2020). The identification of unique neuronal subtypes based on molecular markers gives the opportunity to selectively target neurons intermingled within others in a given area, and describe their behavioral character. We here used gene targeting based on molecular profile and provide evidence that a distinct group of VTA DA neurons characterized by the expression of *NeuroD6* is involved in consummatory and not motivated behavior toward sucrose reward, adding information on the current knowledge of the function of the VTA DA system. Altogether, studies using advanced targeting of isolated DA subpopulations are crucial for understanding the underlying physiology of normal reward-related behavior and for providing a theoretical framework to explain conditions where these processes are compromised, such as drug use disorder, food disorders and depression. By aiming to manipulate only specific neurons implicated in disease, more efficient therapeutic approaches can be developed and unwanted side-effects by targeting the whole DA system can be avoided. Our results provide insights on the role of one of those VTA DA subpopulations in behavior, toward this direction.

## Data availability statement

The raw data supporting the conclusions of this article will be made available by the authors, without undue reservation.

## Ethics statement

The animal study was reviewed and approved by Uppsala University Ethical Committee for Use of Animals in accordance to Swedish and EU legislation.

## Author contributions

ZB conceived planned and performed experiments, analyzed data, and wrote the manuscript (original draft, editing, and revision). GPS and NK performed experiments. ÅW-M: project design, organization and funding, and manuscript editing. All authors reviewed the manuscript, contributed to the article, and approved the submitted version.

## Funding

This work was supported by Uppsala University and by grants to ÅW-M from the Swedish Research Council (Vetenskapsrådet 2017–02039), the Swedish Brain Foundation (Hjärnfonden), Parkinsonfonden, Bertil Hållsten Research Foundation, Zoologiska stiftelsen and Åhlénstiftelsen.

## Conflict of interest

The authors declare that the research was conducted in the absence of any commercial or financial relationships that could be construed as a potential conflict of interest.

## Publisher’s note

All claims expressed in this article are solely those of the authors and do not necessarily represent those of their affiliated organizations, or those of the publisher, the editors and the reviewers. Any product that may be evaluated in this article, or claim that may be made by its manufacturer, is not guaranteed or endorsed by the publisher.
